# Meta-analysis on sex differences in mortality and neurodevelopment in congenital heart defects

**DOI:** 10.1038/s41598-025-92894-w

**Published:** 2025-03-09

**Authors:** Alyssa K. Crain, Zhia N. Lim, Chloe J. Sarfatis, Magela Arias, Travis Holder, Alvaro G. Moreira, Antonio F. Corno, Tina O. Findley

**Affiliations:** 1 Division of Neonatal-Perinatal Medicine, Department of Pediatrics, McGovern Medical School at the University of Texas Health Science Center in Houston and Children’s Memorial Hermann Hospital, Houston, TX 77030 USA; 2 Great Ormond Street Institute of Child Health, University College London, London, UK; 3 Nova Southeastern College of Osteopathic Medicine, Fort Lauderdale, FL USA; 4 Research Medical Library, University of Texas MD Anderson Cancer Center, Houston, TX 77030 USA; 5 Department of Pediatrics, Neonatology Regenerative and Precision Medicine Laboratory, University of Texas Health Science Center at San Antonio, San Antonio, TX USA; 6 Veterans Administration Center for Personalized Medicine, South Texas Veterans Health Care System, San Antonio, TX USA; 7 School of Engineering, University of Leicester, Leicester, UK; 8 Faculty of Science and Engineering, Manchester Metropolitan University, Manchester, UK

**Keywords:** Neonatology, Congenital heart defects, Paediatric research, Cardiology, Risk factors, Neurodevelopmental disorders

## Abstract

Given the increasing survival rates among congenital heart disease (CHD) patients and the growing emphasis on their quality of life, there is a need to comprehensively assess the impact of surgical interventions on neurodevelopmental outcomes. With increasing awareness in sex-related disparities in CHD, there is a need to explore potential differences in surgical mortality and neurodevelopmental outcomes between male and female patients. In this systematic review, we adhered to PRISMA guidelines and PROSPERO registration (#CRD42021225610). Articles published from 2015 to 2021 were searched using MeSH descriptors in three major databases (MEDLINE Ovid, Elsevier Embase, and Cochrane Library). Study selection criteria focused on pediatric (< 18 years of age) CHD patients undergoing primary cardiac surgery. A total of 163 articles that met inclusion criteria were reviewed. The definition and assessment of neurodevelopmental impairment, data extraction, risk of bias assessment, and statistical analysis methods were adhered by blinded reviewers. Previous studies have reported higher rates of early childhood mortality in female patients and higher rates of neurodevelopmental impairment in male patients with CHD requiring surgery. Our meta-analysis suggests that these differences may no longer be valid in contemporary surgical cohorts. However, it is unclear if sex-related risk factors have truly been mitigated with current surgical and medical approaches Our meta-analysis does underscore the need for further research considering sex as a variable and for additional investigative efforts in long term neurodevelopmental outcomes.

**Supplementary Information:**

The online version contains supplementary material available at 10.1038/s41598-025-92894-w.

## Introduction

Congenital heart disease (CHD) is the most prevalent major congenital anomaly worldwide occurring in nearly 1% of live births and contributing significantly to mortality associated with birth defects^1^. Remarkable strides in peri-operative CHD management have resulted in enhanced surgical outcomes and a global decrease in mortality^2,3^. This advancement in medical care has translated into a growing population of CHD survivors, with over 90% now anticipated to reach adulthood,^4^ prompting clinicians and society to shift their focus toward assessing the post-surgery quality of life for these patients.

Neurodevelopmental (ND) impairment is the most significant and frequent sequela of CHD^5^ and correlates with correlates with CHD complexity^6^. Even prior to surgery, patients with CHD have neurobehavioral and neuroimaging abnormalities including hypotonia, microcephaly, brain lesions, and general ND delay^7,8^. Fetal brain injury may be caused by alterations in fetal blood circulation due to structural cardiac abnormalities, particularly during the third trimester^9^. Severe cerebral lesions have been most frequently observed by fetal magnetic resonance imaging among three types of cyanotic CHD: transposition of the great arteries, single ventricle physiology, and hypoplastic left heart syndrome^10,11^. In cases of transposition of the great arteries and single ventricle physiology, oxygen delivery to the brain is reduced because of low oxygen content in the arterial blood. In addition, associated severe abnormalities of the placenta in these cyanotic CHD lesions may also contribute to compromised fetal development^12^.

After birth, patients also face risk of acute neurological events related to CHD surgery such as seizures and strokes, which have been linked to increased likelihood of long-term neurodevelopmental impairment and developmental delays^13^. Consequently, individuals with CHD who have undergone surgical interventions are more likely to require educational support, encompassing tutoring, special education, as well as physical, occupational, and speech therapies^5^. While advancements in peri-operative techniques have improved survival rates, the improvement in neurodevelopmental outcomes has remained relatively modest, with over half of complex CHD patients anticipated to encounter neurodevelopmental disabilities impacting cognitive, language, motor, or social-emotional functions^14^.

More recently, studies have explored underlying demographic differences in post-surgical mortality and the prevalence of ND impairment, with a specific focus on sex differences. While well-documented variations exist between male and female infants in terms of CHD incidence and types^15^, the specific disparities in post-surgery ND impairment rates for male and female patients has not been explored in a more contemporary, international CHD population^16–20^. Considering recent medical and surgical advancements, we conducted a systematic review and meta-analysis to investigate potential sex-related discrepancies in two critical areas: (i) surgical mortality, and (ii) neurodevelopmental outcomes.

## Methods

### Search strategy

The protocol was registered under PROSPERO #CRD42021225610 and adhered to the Preferred Reporting Items for Systematic Review and Meta-Analysis (PRISMA) criteria^21^. With librarian assistance (TFH), we conducted a comprehensive search for articles using three search engines in November 2021: Ovid MEDLINE, Elsevier Embase, and Cochrane Library. Oursearch employed controlled vocabulary subject headings (MeSH and Emtree) related to “congenital heart disease,” “brain injury,” and “neurodevelopment.” We also utilized commands to capture multiple keyword variations to describe several conditions and defects to ensure that the search was both extensive and inclusive. The complete search strategy details can be found in Supplement 1.

To provide a contemporary perspective, we included articles published between 2015 and 2021. This approach enables us to better assess the impact of current strategies. To facilitate review, Rayyan was utilized by blinded reviewers^22^.

### Study selection

In our study selection process, we incorporated the following inclusion and exclusion criteria:

#### Inclusion criteria

1. Patient population included only pediatric patients under 18 years of age.
2. Patient population received primary cardiac surgery for CHD.

#### Exclusion criteria

1. Studies published in languages other than English.
2. Case reports or series with fewer than five patients.
3. Studies missing outcomes of in-hospital mortality or neurodevelopmental impairment.
4. Articles published prior to the year 2015.
5. Abstract-only publications.
6. Studies on cardiac transplantation, catheter-based interventions, or isolated closure of patent ductus arteriosus.

These stringent criteria were applied to ensure the selection of studies that aligned with the scope and objectives of our review, promoting the quality and relevance of the included research.

### Definitions and outcomes

We established our definition of ND impairment in accordance with the Neonatal Research Network’s criteria, which encompassed a range of indicators. These were neurologic examination diagnoses, Bayley Scales of Infant Development III (BSID III) cognitive and motor scores, sensory impairment, and a composite outcome of ND impairment defined by BSID III cognitive score cutoff of < 85 or < 70, BSID III motor score < 70, and the presence of moderate or severe cerebral palsy (CP), bilateral blindness, and hearing impairment^23^.

Our evaluation of publications involved a two-stage process: initial screening titles and abstracts, followed by a comprehensive review of the full text. Independent reviewers, organized into two groups (ZL/MA and AKC/CS), assessed each abstract based on its content. In cases where discrepancies arose between the two reviewers, resolution was achieved through consultation with two authors (TF and AFC) until a consensus was reached.

Subsequently, two reviewers (AFC and TF) independently evaluated the full text of all selected articles, adhering to the same predefined criteria as outlined earlier. Data extracted from the articles included demographic characteristics (race/ethnicity, sex), median age at surgery, mortality rates, and ND assessment. Whenever data on the primary outcome was not disaggregated by sex, efforts were made to contact the original authors for the missing data. Notably, two studies featured overlapping time periods and the same patient cohort, but through communication with the authors, we ensured that data duplication was avoided. These two studies^24,25^ will be jointly presented in our meta-analysis for coherence and accuracy.

### Risk of bias

The articles were scored for internal validity and risk bias using a revised Cochrane risk-of-bias tool for randomized trials (RoB2 tool) and CLARITY tool at McMaster University to assess risk of bias for cohort studies based on the type of study^26,27^ (Table 2). For randomized control trials, factors in determining bias level included randomization of intervention, adequate blinding, treatment of all participants, and data analysis tools pre-determined before study initiation. Low risk of bias randomized trials favor bias towards experimental. Specific questions assessing risk of bias for cohort studies primarily involved assessment of exposure including selection of exposed and non-exposed groups, matching variables, noted prognostic factors. Risk of bias also considered assessment of outcome in the study and adequate follow up of cohorts. Studies with lower risk of bias would then have adequate assessment of exposure and outcomes using objective tools with adequate matching, follow up, and knowledge of prognostic factors or other variables.

### Statistical analysis

Given that both the primary and secondary outcomes were represented as categorical data, number of patients with each outcome relative to the total number of patients were aggregated within the respective groups. Forest plots were employed to display pooled odds ratios (Ors) along with their corresponding 95% confidence intervals (Cis).

To conduct the analyses, DerSimonian and Laird random effects meta-analysis approach was utilized with statistical significance defined as a two-sided *p*-value < 0.05. Recognizing the inherent heterogeneity stemming from variations in participant age ranges, diverse cardiac conditions, procedure timing, and outcome measures across the studies, the need to assess statistical heterogeneity was anticipated. I *I*^*2*^ statistic was used to quantify study heterogeneity, which quantifies the percentage of variability in effect estimates.

To assess the robustness of our findings and identify potential influential studies, we performed a leave-one-out sensitivity analysis using a random-effects model with the Sidik-Jonkman (SJ) estimator for between-study variance. This analysis was conducted by iteratively removing one study at a time and recalculating the pooled odds ratio (OR) and 95% confidence intervals (CI) to determine whether the overall effect size was disproportionately influenced by any single study. In addition, changes in heterogeneity were assessed using I^2^ statistics and tau^2^ estimates to evaluate variations in between-study variance. The results were visualized through a leave-one-out influence plot, where OR values and their corresponding CIs were examined for stability.

For an in-depth evaluation of potential publication bias, qualitative assessments using funnel plots and quantitative assessments via Egger’s linear regression test were utilized. To enable subgroup analyses, a minimum of four articles was considered necessary. All statistical analyses were executed using R, version 4.1.0.

## Results

### Study selection

**Fig. 1:**
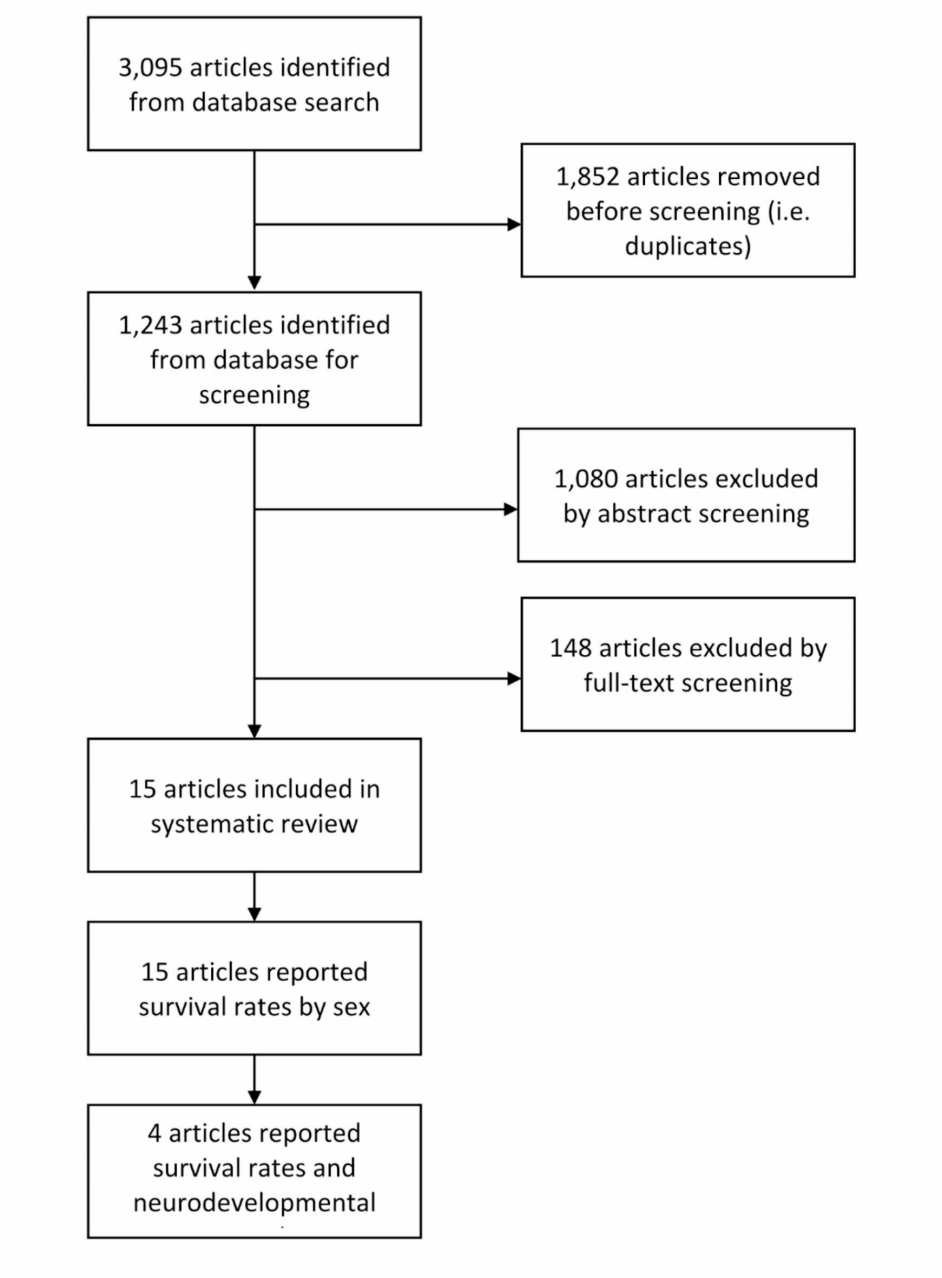
Flow chart of identification of articles and screening for inclusion in meta-analysis.

Figure 1 illustrates the article selection process employed in our meta-analysis. The initial database search yielded a total of 3095 results. Upon removing duplicates, there were 1243 unique results. During the abstract screening phase, 1080 articles were excluded based on predefined criteria. Full texts of the remaining 163 articles were reviewed. Additionally, authors were contacted to provide original data with sex-disaggregated outcomes. Nine of the fifteen of the contacted authors responded to our inquiries. After evaluation of the full texts, an additional 148 articles were excluded based on the exclusion criteria including outcomes irrelevant to the meta-analysis and missing sex-disaggregated data after contacting original authors. Table 1 provides a summary of the 15 studies included in the meta-analysis.

**Table 1:** Summary characteristics of included studies.

Author	Study period	Cardiac phenotype (Age)	Cohort site	Percentage male patients (%)
Atallah	1996–2010	HLHS (7 days–11.5 years)	Stollery Children’s Hospital, Canada	59.5
ElMahrouk	2001–2016	Complex with ECMO (4 days–15 years)	Egypt, Saudi Arabi	59.3
Griffiths	2005–2014	DORV or dTGA (Neonates)	Utah, USA	73.4
Hames	2002–2017	Truncus arteriosus with ECMO (< 60 days)	ELSO Registry	50.6
Hamzah	2002–2017	Truncus arteriosus (< 1 year)	National Inpatient Sample	51.2
Howard	2006–2013	Complex with ECMO (< 29 days)	Boston Children’s Hospital, USA	56.0
Hoxha	2008–2015	Aortic arch hypoplasia, IAA, HLHS (< 30 days)	Verona, Italy	60.7
Lim	2012–2016	Complex (< 10 years)	Singapore	47.0
Manuel	2011–2017	Single ventricle	Angola	51.0
Martin & Cheung	1996–2016	Complex (< 6 weeks corrected age)	Stollery Children’s Hospital, Canada	64.3
Merkle	2008–2016	Complex with ECMO (< 18 years)	Germany	61.5
Newburger	2005–2008	HLHS and Single Ventricle (neonates at surgery, 6 years at follow up)	Single Ventricle Reconstruction Trial	61.9
Szynycer-Taub	2007–2013	Complex with ECMO (< 1 year)	Michigan, USA	58.1
Tomataki	2006–2011	Complex (VLBW) (neonates at surgery, 3 years at follow up)	Japan	33.3

DORV, double-outlet right ventricle; dTGA, d-transposition of great arteries; ECMO, extracorporeal membrane oxygenation; HLHS, hypoplastic left heart syndrome; IAA, interrupted aortic arch; VLBW, very low birth weight.

### Characteristics of included studies

The included studies had diverse geographic distribution, spanning multiple countries, including the United States, Canada, Jordan, Angola, Singapore, Germany, and Italy, with two studies examining international patient cohorts^28,29^. These investigations predominantly adopted a retrospective cohort study design, except for one study that reported data from the Single Ventricle Reconstruction Trial^24^. Notably, the included cohorts varied significantly in study size, study length, geographical location, racial demographics, and number of clinical sites involved.

The articles also exhibited substantial heterogeneity in CHD phenotypes, type of surgical repair, and age at the time of surgery. Among the 15 articles, five studies (33%) focused on patients who required extracorporeal membrane oxygenation (ECMO) following cardiac surgery^28,30–32^. Furthermore, certain studies concentrated on the outcomes of specific cardiac surgical procedures, including the Norwood procedure, first stage of palliative surgery for hypoplastic left heart syndrome^24,33–35^, the arterial switch for transposition of the great arteries or double-outlet right ventricle^36^, truncus arteriosus repair^28,37^, hypoplastic or interrupted aortic arch surgery^38^, or unspecified cardiac surgeries^25,39,40^. Some studies specifically investigated surgical outcomes in patients born premature, who are known to have heightened risk for mortality and neurodevelopmental impairment^25,39^. In aggregate, these 15 articles collectively represented 4999 patients, with a slight male predominance at 54%. Notably, one study reported the largest cohort with 3009 patients^37^ (Table 2).

**Table 2:** Risk of Bias Assessment of studies included in meta-analysis.

Author	Q1	Q2	Q3	Q4	Q5	Q6	Q7	Q8	Total
Howard, T	1	1	1	1	2	1	1	2	10
Griffiths, ER	1	2	1	1	2	1	1	1	10
Hames, DL	1	1	1	1	2	1	3	2	12
ElMahrouk, AF	2	1	1	2	2	1	2	2	13
Hamzah, M	1	2	2	2	2	2	3	2	16
Atallah, J	1	2	2	2	2	1	1	2	13
Tomotaki	1	1	2	2	2	1	2	2	13
Sznycer-Taub	1	1	1	1	2	1	3	2	12
Martin, BJ	1	1	2	2	2	1	2	2	13
Manuel, V	1	2	1	2	2	1	2	2	13
Lim CY	1	1	1	1	2	2	1	1	10
Newburger, JW	1	1	1	1	2	1	1	2	10
Merkle, J	1	1	1	1	2	1	2	1	10
Hoxha, S	1	1	1	2	2	2	2	1	12
Cheung, PY	1	1	2	2	2	1	1	2	12

Q1–Q8 defined as in the CLARITY risk of bias tool for cohort studies^27^.

Please see Supplemental Table 1 for the questions and scoring.

### Primary outcomes of interest

#### Mortality

**Fig. 2:**
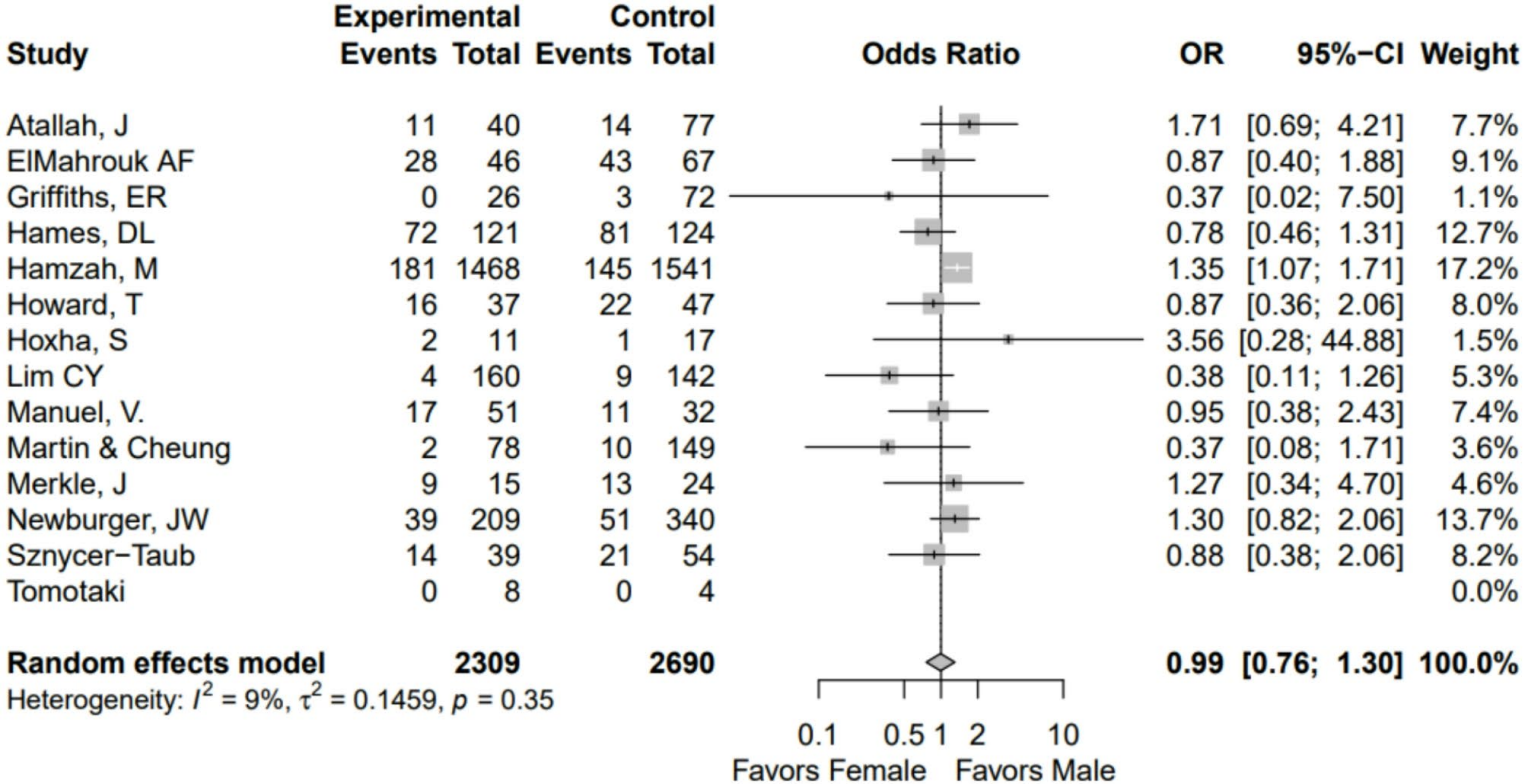
Forest plot for comparison of mortality after surgery for congenital heart defect.

Sex-disaggregated mortality data was available in all included articles. The overall sex distribution aligned with the expected pattern, as many of the cardiac phenotypes examined exhibited a male predominance^41,42^. Combined rates of all mortality were 424/2690 (15.8%) in male CHD patients and 398/2309 (17.2%) in female CHD patients. No difference was observed in mortality by sex (OR 0.99, 95% CI 0.76–1.30) (Fig. 2). No publication bias was observed on the funnel plot (Supplemental Fig. 1).

**Fig. 3:**
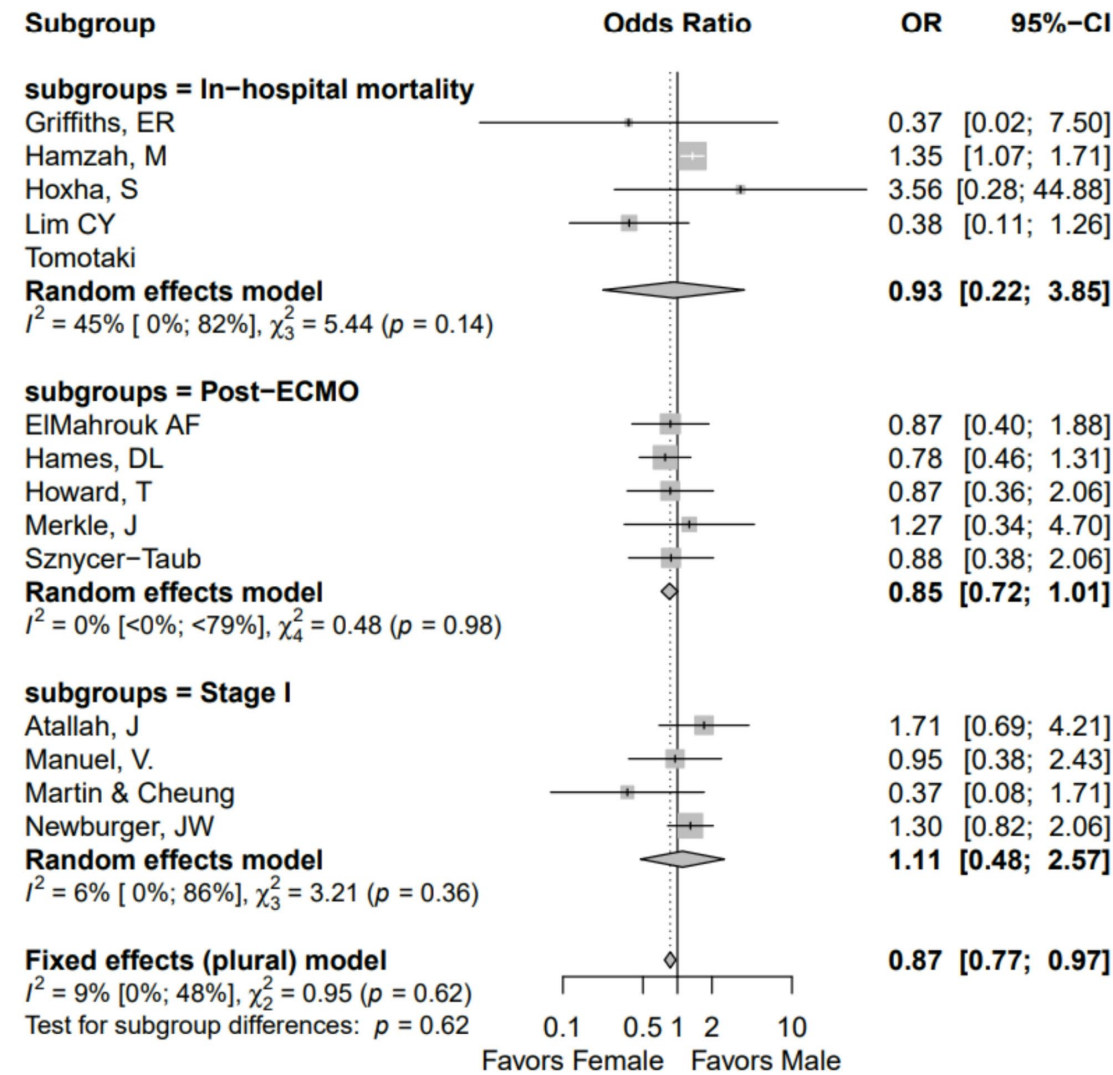
Forest plot of mortality after primary cardiac repair showing sex difference in mortality by sub-groups.

Studies varied in reported timing of mortality, so mortality rates were also analyzed by sub-groups: in-hospital mortality (n = 5 studies), post-ECMO mortality (n = 5 studies), and post-Stage I mortality (n = 4 studies). This analysis did show females had overall decreased odds for mortality (OR 0.85; 95% CI 0.77–0.97), but no significant difference in mortality for male or female patients in each of the sub-groups (Fig. 3).

#### Neurodevelopment outcomes

Only three studies included ND outcomes in male and female patients after surgery for CHD. Two of the articles assessed children with the BSID-III with BSID-III assessments, defining a cognitive score less than 70 as severe NDI^24,25^. These two studies had overlapping cohorts, so results were merged and duplicates removed for the meta-analysis. The Single Ventricle Reconstruction Trial assessed children with the BSID-II^33^. BSID-II Motor Development scores less than 70 was defined as severe NDI. In another study children were assessed with the Kyoto Scale of Psychological Development, which has been well-correlated to BSID^39^. The article defined a developmental quotient (DQ) score less than 70 as severe NDI^36^.

**Fig. 4:**
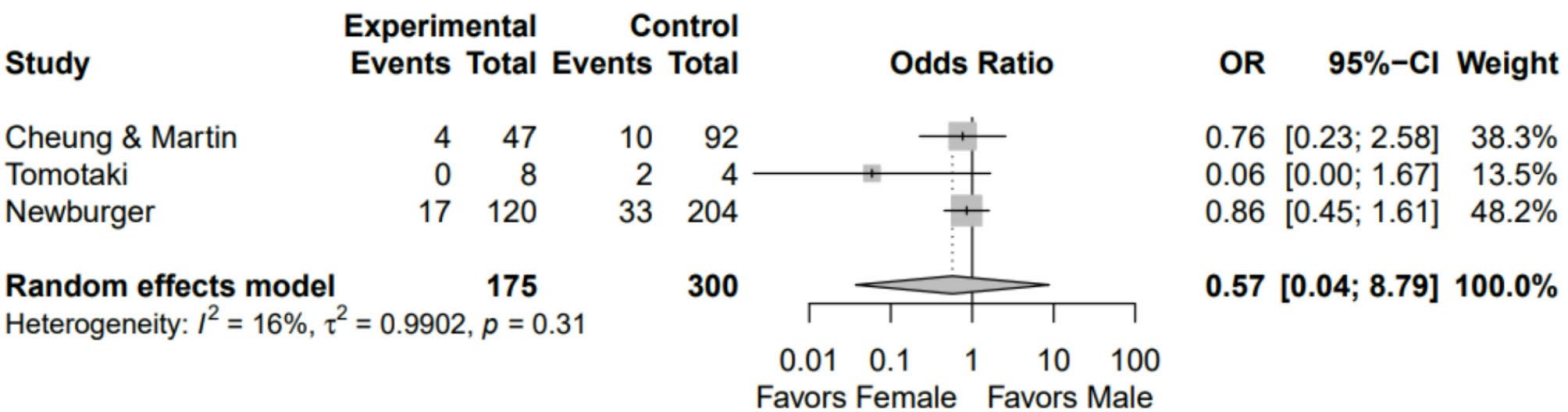
Forest plot of neurodevelopmental impairment after primary cardiac repair showing no significant sex differences in neurodevelopmental impairment.

A total of 475 patients had neurodevelopmental outcomes available, 63% male and 37% female. Rates of severe NDI after surgery for congenital heart defects were 15% in male patients and 12% in female. No sex differences were noted for this outcome (Fig. 4). No publication bias was observed on the funnel plot (Supplement 2).

### Methodological quality (risk of bias)

All the studies included in this analysis were cohort studies, and the CLARITY tool was employed to assess for risk of bias^27^. This comprehensive tool evaluates the potential sources of bias in cohort studies across eight distinct domains, which encompass study participation, study attrition, measurement of prognostic factors, outcome measurement, management of study confounding, the rigor of statistical analysis, intervention integrity, and an overall assessment of the risk of bias. Each domain is scored using a Likert scale, with four possible choices ranging from low risk (1) to high risk (4). Consequently, a study that receives all low-risk scores across all domains would accumulate a total score of eight, while the maximum attainable score is 32.

Total scores obtained by the reviewed articles fell within the range of 10–16, indicating varying degrees of potential bias across the included studies. Notably, the three domains with the highest observed risk of bias were related to the measurement of prognostic factors, the management of study confounding, and the assessment of follow-up outcomes. Egger’s test for funnel plot asymmetry did not indicate significant publication bias (t = − 0.45, df = 14, *p* = 0.6596), suggesting no strong evidence of small-study effects in the meta-analysis.

## Discussion

Our meta-analysis of 15 studies did not show sex differences in mortality or ND impairments in children following cardiac surgery. Studies involved surgeries performed between 1996 and 2017, and included studies represented a global pediatric population. These factors increased the relevance and generalizability of our study. Overall, the results of our meta-analysis suggest previously reported sex differences in death and ND impairment following surgery for CHD were not present in a more contemporary and globally-represented population of pediatric patients^16,42–47^. We did note a trend towards higher mortality in female patients in several of the studies included in our meta-analysis^24,35,40^, which is consistent with older studies investigating the effects of sex on 30-day in-hospital mortality following pediatric cardiac surgeries performed in the United States from 1982 to 2007^16^. However, contradictory results were reported by another older study, showing no sex differences in mortality in pediatric cardiac surgeries reported between 2007 and 2009 in the United States^48^.

Despite our findings, sex may still be an important biologic risk factor in pediatric surgical outcomes. The effect of sex has been well-described in adult cardiac surgeries including valvular and coronary artery surgeries with many studies demonstrating the higher likelihood of adult women developing heart failure with higher risk of associated mortality^49–54^. Additionally, there are well-documents sex differences in outcomes for adult patients with medical conditions affecting the cardiovascular system such as diabetes, systemic hypertension, cholesterol and triglyceride dysregulations^55–58^. There are behavioral factors and clinical biases known to increase these risks in women like delayed recognition of disease^59,60^. However, there is also evidence of chromosomal and gonadal hormone factors driving sex-dependent responses to cardiac ischemic/hypoxic injury^61–63^. Analysis of a possible correlation between genetic malformations, type of congenital heart defects, and related outcomes for surgery and ND outcomes could be more insightful as mortality and ND outcomes vary considerably depending on if patients have an underlying genetic condition for example. However, considering the huge spectrum of congenital heart defects in a relatively small patient population, this analysis would require extensive epidemiological studies, performed with prospective sequential multi-center data collection. This would be another area of research that would provide more evidence for any difference in outcomes between sexes.

While cardiac disease in the pediatric population is more often structural, similar sex-related mechanisms may still be relevant. The National Institute of Health (NIH) has required the inclusion of sex as a variable in all NIH-funded studies since 2016^64^. In our literature review, many studies published between 2016 and 2021 were excluded due to lack of sex-disaggregated outcomes. Still, sex bias persists in human surgical clinical research in sex-based reporting, analysis, and discussion of data, with cardiac surgery having the highest rates of sex bias^65^. Another research bias is lower female enrollment and study participation in medical specialties including pediatric and cardiac-related clinical trials^66^.

As survival rates continues to improve, research interest has focused on quality of life, enhancing the ND outcomes of children with congenital heart defects. Multiple factors including underlying genetic conditions, chronic fetal and neonatal hypoxia, cardiopulmonary bypass, and psychosocial stressors increase the risk for ND impairments^6^. Reporting of neurological sequelae has been inconsistent and often limited due to acute post-operative injuries such as strokes and seizures and partially due to better recognition and detection of early, pre-operative brain injury^67^. As a result, studies only report longer-term ND outcomes with only infrequently validated assessments, not necessarily correlated with the time and the cause of the neurological injury. In our systematic review, only five studies reported mortality and ND outcomes using a validated assessment tool, and one was excluded from the ND meta-analysis because the data was not sex-disaggregated^31^. The ability to detect any sex differences in ND impairments in the included studies were hampered by the small sample size.

### Limitations of the study

Study limitations include the heterogeneity of the studies in the outcomes of interest, mainly timing of mortality and method of neurodevelopmental assessment. Neurodevelopmental assessments included the BSID-II, BSID-III, and the Kyoto Scale of Psychological Development. Each tool has been validated, but their correlation between each other continues to be a source of debate. While in our study, ND results were derived from all three of these assessments, the differences could have affected our outcomes in how many patients had evidence of ND impairment. Another limitation was the variety of cardiac phenotypes included in the meta-analysis, each with different expected rates of adverse events and complications. However, we limited the studies to primary cardiac surgeries requiring cardiopulmonary bypass, excluding minor procedures. Additionally, the studies had a variety of patient populations with different possible confounders including socioeconomic status, genetic syndromes that were not able to be included in the meta-analysis. Lastly, the meta-analysis had a relatively small sample size and may have been underpowered to detect a difference. There is a specific risk of Type II error, given the limited sample size in some included studies. While the pooled effect estimates did not reach statistical significance, this may reflect insufficient statistical power rather than a true absence of effect. To mitigate this concern, we conducted a leave-one-out sensitivity analysis, which confirmed that no single study disproportionately influenced the overall results (Supplemental Fig. 3). However, the possibility remains that true differences were not detected due to sample size constraints. While there was a small number of studies reporting sex-disaggregated data, a concerted effort was made to contact original authors in order to include as many studies as possible. Future studies with larger datasets and improved statistical power are warranted to validate these findings.

## Conclusion

Previous studies have reported higher rates of early childhood mortality in female patients and higher rates of neurodevelopmental impairment in male patients with CHD requiring surgery. Our meta-analysis including a more contemporary cohort and international study sites suggests that these differences may no longer be valid. However, it is unclear if sex-related risk factors have truly been mitigated with current surgical and medical approaches. Despite the limitations of our study, our comprehensive literature review highlights the imperative for further research to incorporate sex as a variable. Larger, prospective studies would allow for better elucidation of differences between sexes and description of any hormonal or genetic factors. Additionally, our results underscore the critical need for heightened rigor in examining neurodevelopmental outcomes in future research pertaining to CHD.

## Electronic supplementary material

Below is the link to the electronic supplementary material.

Supplementary Material 1

Supplementary Material 2

Supplementary Material 3

Supplementary Material 4

Supplementary Material 5

## Data Availability

The data generated from the articles included in our meta-analysis and our results are included in this published article. Complete datasets or additional information are available from the corresponding author on request.
