# Supplementary material for: Meta-analysis on sex differences in mortality and neurodevelopment in congenital heart defects
Source: Sci Rep. 2025 Mar 9;15:8152. doi: 10.1038/s41598-025-92894-w (PMC11891313; doi:10.1038/s41598-025-92894-w)
Supplement: Supplementary file 2 — Supplementary Material 2 [file 41598_2025_92894_MOESM2_ESM.docx]

**SUPPLEMENTAL FIGURE 2:** Funnel plot of studies examining sex differences in neurodevelopmental impairment after primary cardiac repair

**
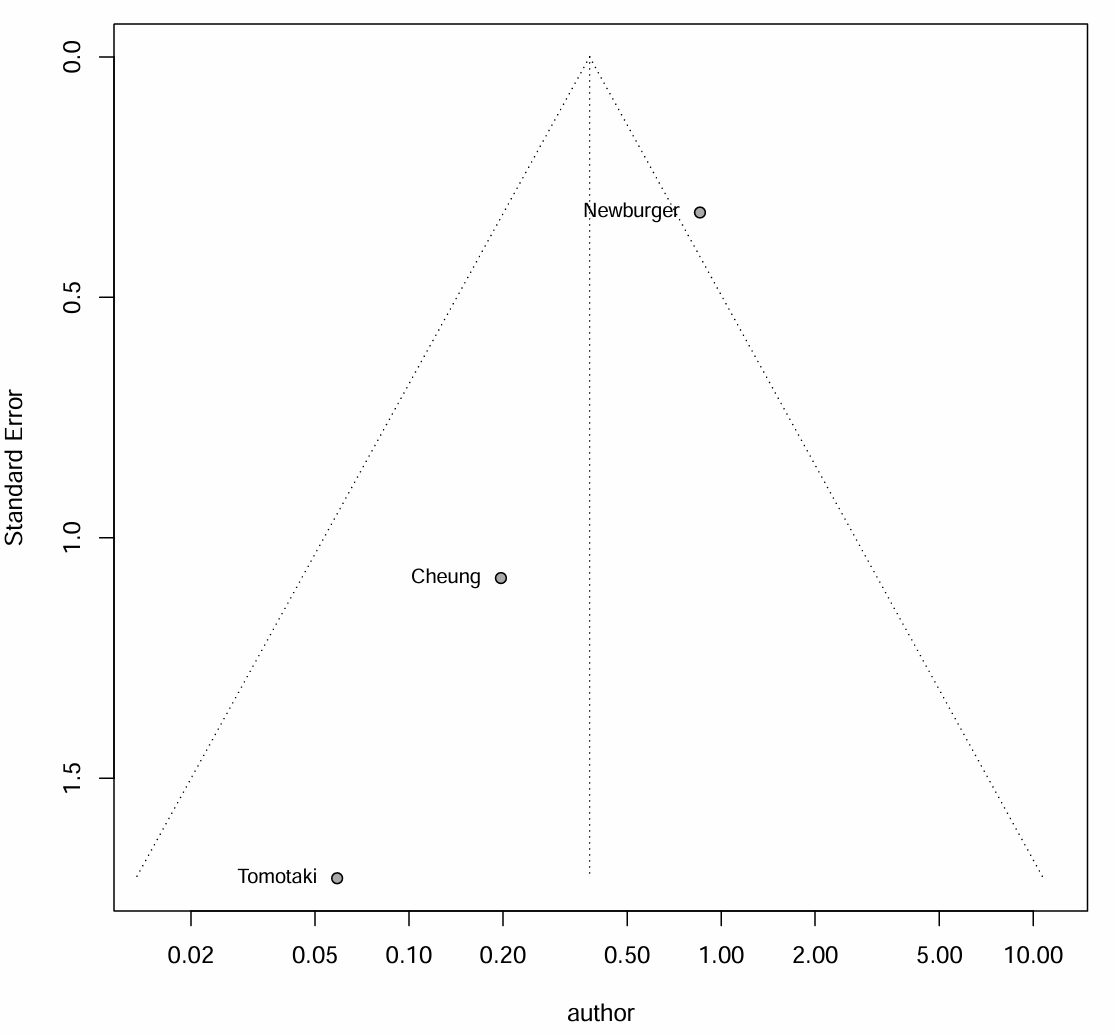
**
