# Supplementary figures and images for: Meta-analysis on sex differences in mortality and neurodevelopment in congenital heart defects
Source: Sci Rep. 2025 Mar 9;15:8152. doi: 10.1038/s41598-025-92894-w (PMC11891313; doi:10.1038/s41598-025-92894-w)

**SUPPLEMENTAL FIGURE 3: Leave-one-out sensitivity analysis to assess influences of each study**

**
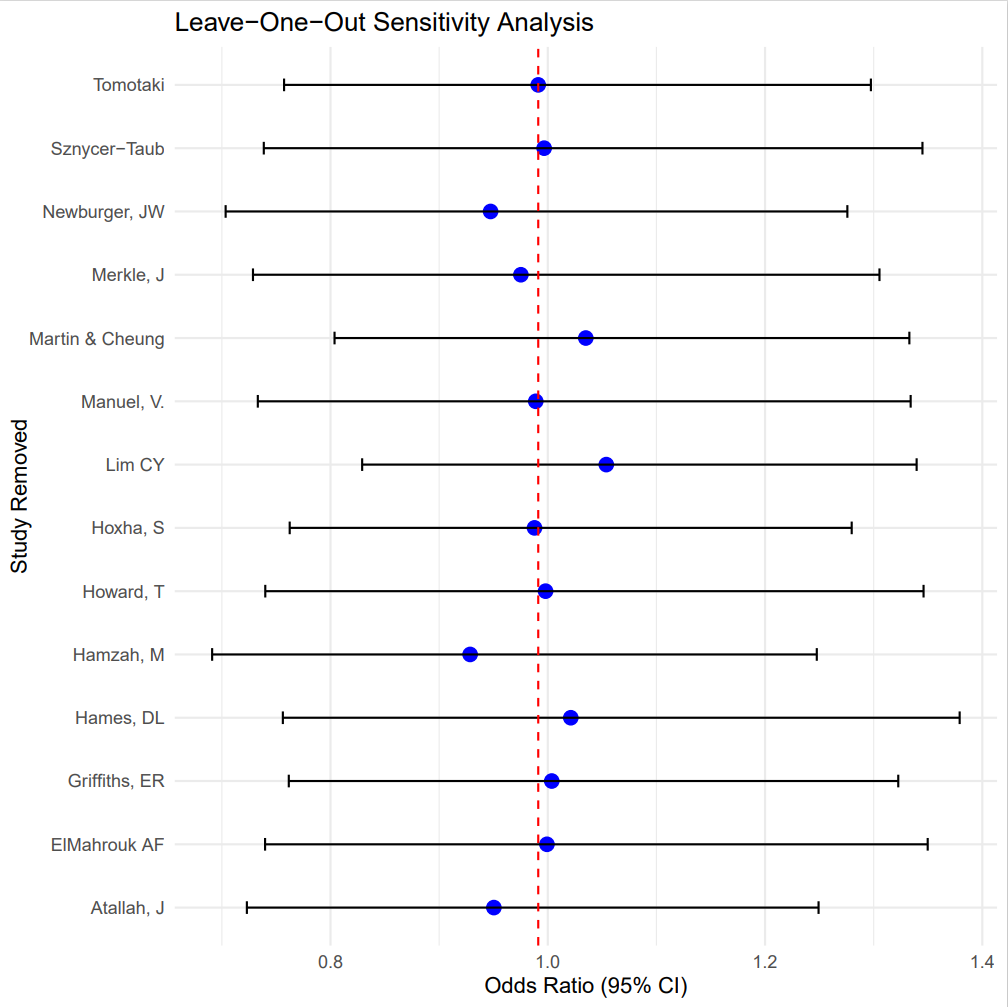
**

Supplement: Supplementary file 3 — Supplementary Material 3 [file 41598_2025_92894_MOESM3_ESM.docx]
