# Supplementary material for: Meta-analysis on sex differences in mortality and neurodevelopment in congenital heart defects
Source: Sci Rep. 2025 Mar 9;15:8152. doi: 10.1038/s41598-025-92894-w (PMC11891313; doi:10.1038/s41598-025-92894-w)
Supplement: Supplementary file 4 — Supplementary Material 4 [file 41598_2025_92894_MOESM4_ESM.docx]

**Ovid Medline**

#1 exp Heart Defects, Congenital/ or Heart Diseases/cn or ((aortopulmonary or aorticopulmonary or atrial or cardiac or cardio* or coronary or heart or intraventricular or septal* or ventricular) adj3 (abnormal* or anomal* or congenital or defect* or malform*)).ab,kw,ti.

#2 exp Infant/ or ("babies" or "baby" or "infan*" or "neonat*" or "new born*" or "newborn*").ab,kw,ti.

#3 exp Mortality/ or (death* or fatal* or mortalit*).ab,kw,ti.

#4 Brain Diseases/ or exp Auditory Diseases, central/ or Brain Injuries, Diffuse/ or exp Brain Injury, Chronic/ or Cerebral Palsy/ or exp Cerebrovascular Disorders/ or Developmental Disabilities/ or exp Hypoxia, Brain/ or (neurodevelopment* or spastic diplegia*).ab,kw,ti.

#5 (((brain or cerebral or cerebrovascular or development* or encephalopath* or "intracranial vascular" or intellectual* or mental* or neurolog*) adj1 (abilit* or anoxi* or delay* disabilit* or dysfunct* or hypoxi* or impair* or insufficien* or pals* or retard*)) or (auditory adj2 (disease* or disorder* or dysfunct*))).ab,kw,ti.

#6 4 or 5

#5 1 and 2 and 3 and 6

**Elsevier Embase**

#1 'infant'/exp OR 'babies' OR 'baby' OR 'infan*' OR 'neonat*' OR 'new born*' OR 'newly born*' OR 'newborn*':ab,kw,ti

#2 'congenital heart malformation'/exp OR 'congenital heart disease'/exp OR ((aortopulmonary OR aorticopulmonary OR atrial OR cardiac OR cardio* OR coronary OR heart OR intraventricular OR septal* OR ventricular) NEAR/3 (abnormal* OR anomal* OR congenital OR defect* OR distress* OR malform*))

#3 'mortality'/de OR 'childhood mortality'/de OR death:ab,kw,ti OR fatal*:ab,kw,ti OR mortalit*:ab,kw,ti

#4  'brain disease'/de OR 'brain hypoxia'/exp OR 'brain injury'/exp OR 'cerebral palsy'/exp OR 'cerebrovascular disease'/exp OR 'developmental disorder'/exp OR 'diffuse brain injury'/exp OR 'perception deafness'/exp OR neurodevelopment* OR 'spastic diplegia*'

#5 ((brain OR cerebral OR cerebrovascular OR development* OR encephalopath* OR 'intracranial vascular' OR intellectual* OR mental* OR neurolog*) NEXT/1 (abilit* OR anoxi* OR delay* OR disabilit* OR dysfunct* OR hypoxi* OR impair* OR insuffcien* OR pals* OR retard*)):ab,kw,ti

#6 #4 OR #5

#7 #1 AND #2 AND #3 AND #6

**Cochrane Library**

#1 MeSH descriptor: [Heart Defects, Congenital] explode all trees

#2 MeSH descriptor: [Heart Diseases] explode all trees and with qualifier(s): [congenital - CN]

#3 ((aortopulmonary or aorticopulmonary or atrial or cardiac or cardio* or coronary or heart or intraventricular or septal* or ventricular) NEAR/3 (abnormal* or anomal* or congenital or defect* or malform*))

#4 #1 OR #2 OR #3

#5 MeSH descriptor: [Infant] explode all trees

#6 'babies' or 'baby' or 'infan*' or 'neonat*' or 'new born*' or 'newborn*'

#7 #4 OR #5

#8 MeSH descriptor: [Brain Diseases] this term only

#9 MeSH descriptor: [Auditory Diseases, Central] explode all trees

#10 MeSH descriptor: [Brain Injuries, Diffuse] this term only

#11 MeSH descriptor: [Brain Injury, Chronic] 4 tree(s) exploded

#12 MeSH descriptor: [Cerebral Palsy] explode all trees

#13 MeSH descriptor: [Cerebrovascular Disorders] explode all trees

#14 MeSH descriptor: [Developmental Disabilities] explode all trees

#15 MeSH descriptor: [Hypoxia, Brain] explode all trees

#16 neurodevelopment* or 'spastic diplegia*'

#17 ((brain or cerebral or cerebrovascular or development* or encephalopath* or 'intracranial vascular' or intellectual* or mental* or neurolog*) NEAR/1 (abilit* or anoxi* or delay* disabilit* or dysfunct* or hypoxi* or impair* or insufficien* or pals* or retard*))

#18 (auditory) NEAR/2 (disease* or disorder* or dysfunct*)

#19 #8 OR #9 OR #10 OR #11 OR #12 OR #13 OR #14 OR #15 OR #16 OR #17 OR #18

#20 MeSH descriptor: [Mortality] explode all trees

#21 death* or fatal* or mortalit*

#22 #20 OR #21

#23 #4 AND #7 AND #19 AND #22
