# Supplementary material for: Meta-analysis on sex differences in mortality and neurodevelopment in congenital heart defects
Source: Sci Rep. 2025 Mar 9;15:8152. doi: 10.1038/s41598-025-92894-w (PMC11891313; doi:10.1038/s41598-025-92894-w)
Supplement: Supplementary file 5 — Supplementary Material 5 [file 41598_2025_92894_MOESM5_ESM.docx]

**SUPPLEMENTAL TABLE 1: Questions and scores for the CLARITY risk of bias tool as seen in TABLE 2^27^.**

| **QUESTION** | **SCORE** | **SCORE** | **SCORE** | **SCORE** |
| --- | --- | --- | --- | --- |
| Q1:Was selection of exposed and non-exposed cohorts drawn from the same population? | Definitely yes (low risk of bias) = 1 | Probably yes =2 | Probably no = 3 | Definitely no (high risk of bias) = 4 |
| Q2: Can we be confident in the assessment of exposure? | Definitely yes (low risk of bias) = 1 | Probably yes =2 | Probably no = 3 | Definitely no (high risk of bias) = 4 |
| Q3: Can we be confident that the outcome of interest was not present at start of study? | Definitely yes (low risk of bias) = 1 | Probably yes =2 | Probably no = 3 | Definitely no (high risk of bias) = 4 |
| Q4: Did the study match exposed and unexposed for all variables that are associated with the outcome of interest or did the statistical analysis adjust for these prognostic variables? | Definitely yes (low risk of bias) = 1 | Probably yes =2 | Probably no = 3 | Definitely no (high risk of bias) = 4 |
| Q5: Can we be confident in the assessment of the presence or absence of prognostic factors? | Definitely yes (low risk of bias) = 1 | Probably yes =2 | Probably no = 3 | Definitely no (high risk of bias) = 4 |
| Q6: Can we be confident in the assessment of outcome? | Definitely yes (low risk of bias) = 1 | Probably yes =2 | Probably no = 3 | Definitely no (high risk of bias) = 4 |
| Q7: Was the follow up of cohorts adequate? | Definitely yes (low risk of bias) = 1 | Probably yes =2 | Probably no = 3 | Definitely no (high risk of bias) = 4 |
| Q8: Were co-interventions similar between groups? | Definitely yes (low risk of bias) = 1 | Probably yes =2 | Probably no = 3 | Definitely no (high risk of bias) = 4 |
